# Post-infectious inflammatory response syndrome in an HIV-negative patient after *Cryptococcus gattii* meningoencephalitis: a case report and review of the literature

**DOI:** 10.1186/s13256-023-04066-x

**Published:** 2023-08-05

**Authors:** Jianhua Lan, Luyi Lv, Ling Ye, Tao Wang, Zhiyu Wu, Shugen Wu, Chunxian Peng, Weili Lu, Tao Lu

**Affiliations:** 1 The 2nd Clinical Medical College of Zhejiang Chinese Medical University, Hangzhou, 310053 Zhejiang Province China; 2 Department of Infectious Diseases, The Quzhou Affiliated Hospital of Wenzhou Medical University, Quzhou People’s Hospital, Quzhou, 324000 Zhejiang Province China

**Keywords:** Mycosis, *Cryptococcusgattii*, Inflammatory response syndrome, Meningoencephalitis

## Abstract

**Background:**

Cryptococcal meningitis (CM) is an inflammatory mycosis of the central nervous system caused by meninge infection or brain parenchyma with *Cryptococcus* species. It is associated with high morbidity and mortality, and patients with acquired immune deficiency syndrome are particularly susceptible. There have been increasing reports of CM in HIV-negative patients in China over the last few years.

**Case presentation:**

A 31-year-old healthy Chinese male presented with fever and gradually developed headache, projectile vomiting, and other manifestations that were later confirmed as *Cryptococcus gattii* meningoencephalitis. However, multiple disease changes occurred during the course of treatment, and the regimen was accordingly modified after the diagnosis of post-infectious inflammatory response syndrome (PIIRS). The patient eventually recovered.

**Conclusion:**

There has been a growing trend in the incidence of *C. gattii* meningoencephalitis in HIV-negative patients. It shows rapid onset and severe prognosis. This case report can provide a reference to treat PIIRS following CM in HIV-negative patients.

## Background

Cryptococcal meningitis (CM) is an inflammatory mycosis of the central nervous system caused by meninge infection or brain parenchyma with *Cryptococcus* species [1]. It is associated with high morbidity and mortality, and is particularly common in patients with acquired immune deficiency syndrome. There has been a growing trend in the incidence of *C. gattii* meningoencephalitis in HIV-negative patients in China [2]. It is characterized by rapid onset and poor prognosis, and the occurrence of post-infectious inflammatory response syndrome (PIIRS) during treatment worsens patient outcomes. The present case report provides a reference to treat HIV-negative patients that develop PIIRS during the treatment for CM.

## Case presentation

**Fig. 1:**
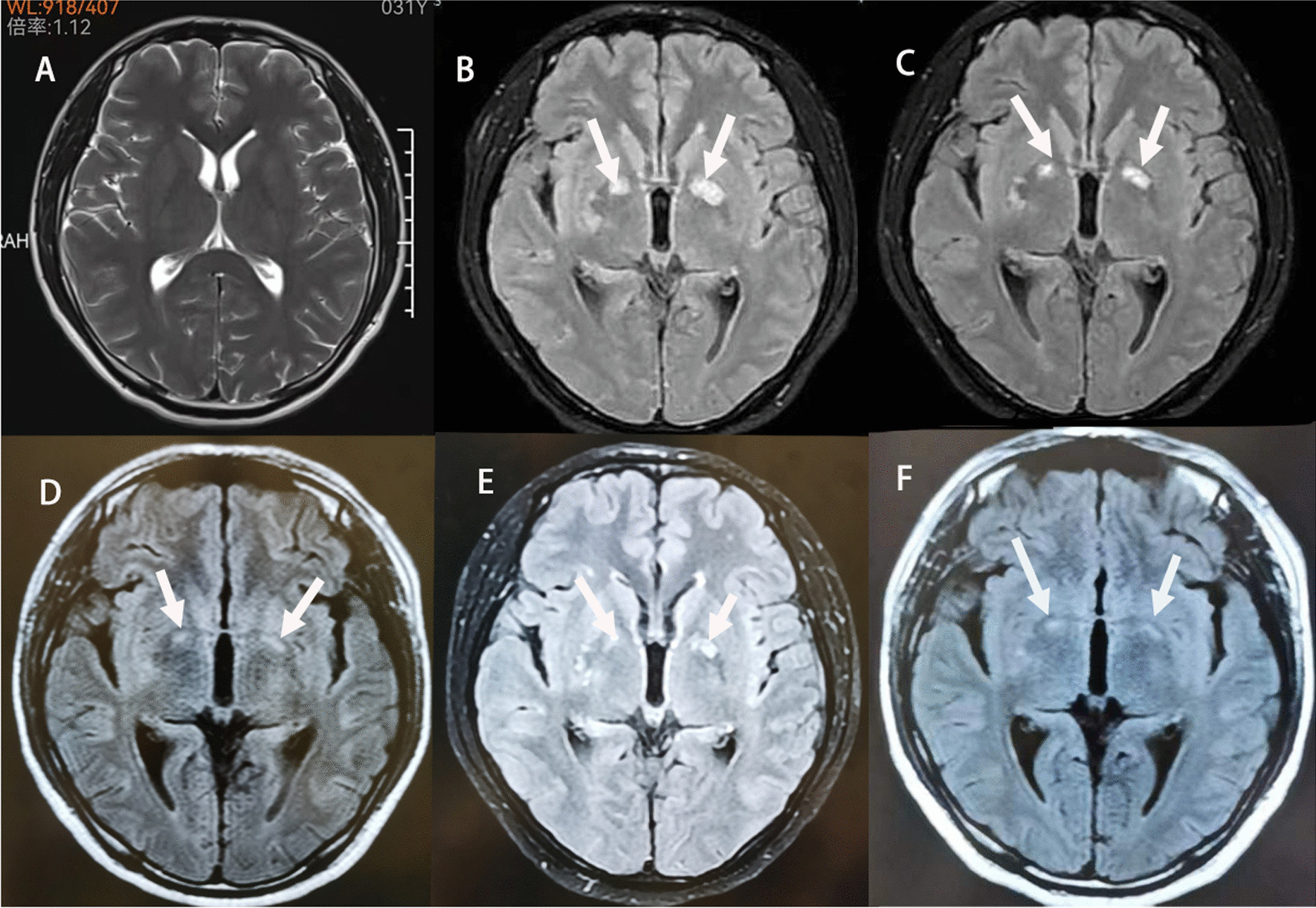
Cranial MRI images **A**–**F**. **A** January 28, 2022—no significant patchy shadows in the bilateral internal capsules and the right basal ganglia. **B** February 25, 2022—patchy abnormal shadows in bilateral internal capsules and right basal ganglia (arrows), which was diagnosed as Cryptococcal infection. **C** March 14, 2022—smaller patchy abnormal shadows in bilateral internal capsules and right basal ganglia (arrows). **D** May 17, 2022—patchy shadows in bilateral internal capsules and the right basal ganglia (arrows). The lesions were smaller than before. **E** July 6, 2022—patchy shadows in bilateral internal capsules and the right basal ganglia (arrows). The lesion in the right basal ganglia was slightly smaller than before, and the left lesion was roughly similar as before. **F** September 25, 2022—patchy shadows in bilateral internal capsules and the right basal ganglia (arrows). The lesions were significantly smaller than before. Note: Magnetic resonance imaging

The patient was a 31-year-old Chinese male logger. He was in good health and was from Xiangyang city, Hubei Province, China. The patient was admitted to the hospital on January 31, 2022 after experiencing fever for 1 week (body temperature up to 38.6 ℃) and severe headache (primarily in the forehead) for 3 days. He had also experienced nausea and projectile vomiting several times. These symptoms had not improved after oral medication (details not known), and the patient presented with fever on admission. He underwent lumbar puncture at a local hospital for cerebrospinal fluid (CSF) examination. The CSF pressure was 350 mmH_2_O (1 mmH_2_O = 0.0098 kPa, normal 80–180 mmH_2_O), and the cell count was 118/µL. Biochemical analysis of the CSF revealed 2.15 mmol/L glucose, 119.4 mmol/L chloride, and 0.81 g/L albumin. The cranial MRI showed small ischemic lesions in the bilateral frontal lobes, and meningitis was suspected. The symptoms did not improve after injections of ganciclovir, 20% mannitol, and furosemide. The patient was transferred to the emergency department of our hospital on January 31, 2022. Since the cranial CT scan showed no significant abnormalities, he was eventually admitted to the neurology department. Physical examination on admission revealed a temperature of 36.3 °C, clear consciousness but weak spirit, neck resistance, two fingers of the chin to chest distance, equally rounded pupils about 3 mm in diameter, reactive to light muscle strength of level 5 in the four limbs, normal muscle tone, and negative Brudzinski’s sign. The results of blood test were as follows: 9.9 × 10^9^/L white blood cells (WBC, normal (4–10) × 10^9^/L), 143 g/L hemoglobin (Hb, normal 130-175 g/L), 363 × 10^9^/L platelets (PLT, normal (100–300) × 10^9^/L), 44.16 mg/L C-reactive protein (CRP, normal 0-5 mg/L), 0.62 mg/LFEU D-dimer, 14.9 s prothrombin time (PT), 0.031 ng/mL procalcitonin (PCT, normal < 0.05 ng/mL), 39.9 U/L alanine aminotransferase (ALT, normal 4–48 U/L), 13.5 U/L aspartate aminotransferase (AST, normal 4–42 U/L), 68.5U/L alkaline phosphatase (AKP, normal 34–121U/L), 102.4U/L glutamyl transpeptidase (GGT, normal 4–60U/L), 154.2U/L α-hydroxybutyrate dehydrogenase (HBDH, normal 74–199U/L), 37.2U/L creatine kinase (CK, normal 22–269U/L), 191.6U/L lactate dehydrogenase (LDH, normal 109–245U/L), 32.2 × 10^6^/L CD4^+^T lymphocytes, negative anti-HIV antibody, and negative syphilis antibody. Based on these findings, intracranial infection was the first diagnosis, with cryptococcal meningitis (CM), tuberculous meningitis, or viral meningitis as the possibilities. The patient was given two intravenous injections of 0.25 g ganciclovir and one of 2 g ceftriaxone sodium on 31 January. Furthermore, 250 ml 20% mannitol was administered three times daily, and 250 ml glycerol fructose was administered twice daily to reduce intracranial pressure. Therapeutic lumbar puncture was performed several times to drain the CSF and relieve intracranial hypertension (Table 1). On February 2, the lumbar puncture revealed CSF pressure of more than 400 mmH_2_O and a positive ink stain. The patient was transferred to our department on February 3 and diagnosed with CM following consultation. Therefore, ganciclovir and ceftriaxone sodium were discontinued, and the treatment was switched to intravenous injection of 35 mg amphotericin B daily (progressively increased to 0.25 mg/kg twice daily) and 1.5 g oral fluorocytosine four times daily. Lumbar puncture was again performed on February 6, and the CSF pressure was 200 mmH_2_O, with positive ink stain, negative GeneXpert, and positive for *Cryptococcus* capsular antigen (titer of 1:1280). The CSF was positive for *Cryptococcus* culture on February 8, and the drug sensitivity test was positive for amphotericin B, 5-fluorocytosine, fluconazole, itraconazole and voriconazole. The symptoms of fever, headache, orbital pain, or vomiting ceased after the treatment, and the CSF pressure steadily decreased and the *Cryptococcus* culture was negative multiple times. However, the patient developed headache and projectile vomiting on February 21, and lumbar puncture revealed CSF pressure of more than 400 mmH_2_O, positive ink stain, and negative for *Cryptococcus* capsular antigen (titer of 1:640). Contrast-enhanced cranial MRI showed patchy shadows in bilateral internal capsules and the right basal ganglia on February 25, and cryptococcal infection was considered (Fig. 1). Due to the possibility of PIIRS, 5 mg dexamethasone sodium phosphate injection was added to the intensive anti-cryptococcal therapy. The patient no longer experienced headaches, orbital pain or vomiting. Based on these findings, a definitive diagnosis of CM was made. Contrast-enhanced cranial MRI on March 14 showed patchy shadows in bilateral internal capsules and right basal ganglia, which were smaller than previously observed. Therefore, dexamethasone sodium phosphate injection was discontinued and anticryptococcal treatment was continued. The patient started to experience headache and orbital discomfort on March 21, which were alleviated once 5 mg sodium phosphate dexamethasone was re-initiated. The treatment was switched to 28 mg oral methylprednisolone daily on March 23, and the patient did not experience any apparent discomfort. The patient was discharged on March 29 with instructions to take 800 mg oral fluconazole and 24 mg oral methylprednisolone.

**Table 1:** Cerebrospinal fluid test

Date	Pressure (mmH_2_O)	Ink stain	Karyocyte (cell/uL)	Percentage of neutrophils (%)	Lymphocyte percentage (%)	Protein content (0.20–0.40 g/L)	Glucose (2.50–4.40 mmol/L)	Chloride (120.0–130.0 mmol/L)	Lactate dehydrogenase (3.0–40.0U/L)	Adenosine deaminase (4.0–24.0U/L)	Culture	titers
January 28	350	–	118	35.0	60.0	0.81	2.15	119.4	34.2	/	–	
February 2	> 400	+	300	35.0	60.0	0.70	1.25	120.6	36.2	1.2	+	
February 6	90	+	55	36.0	59.0	0.80	1.01	120.3	31.8	1.1	+	1:1280
February 10	270	+	5	–	–	0.60	1.59	122.5	21.2	1.6	–	–
February 16	175	+	80	2.0	93.0	0.70	2.26	124.1	20.7	1.2	–	–
February 21	> 400	+	100	2.0	98.0	0.50	2.46	121.4	17.3	0.3	–	1:640
February 24	290	+	55	6.0	82.0	0.80	1.48	122.2	16.3	0.5	–	-
March 1	160	+	95	2.0	96.0	0.70	2.83	125.1	15.3	0.5	–	1:320
March 7	130	+	20	–	–	0.70	3.08	120.6	16.9	0.5	–	–
March 13	150	+	15	–	–	0.50	3.29	124.6	15.3	0.3	–	1:160
March 20	350	+	70	2.0	96.0	0.40	2.18	121.3	15.4	0.1	–	1:160
March 27	200	+	30	–	–	0.40	3.15	128.4	14.7	0.3	–	-
April 14	180	–	5	–	–	0.50	3.00	125.7	14.9	0.3	–	–
May 17	230	–	5	–	–	0.34	2.90	130.7	14.6	2.3	–	1:80
July 4	300	–	10	–	–	0.46	2.47	125.4	13.3	-	–	–
September 27	250	–	0	–	–	0.36	3.29	121.8	13.0	1.7	–	1:20

The patient was regularly followed-up, and the methylprednisolone was gradually reduced by 4 mg every 2 weeks. The patient ceased to take methylprednisolone on July 2, and developed headache and orbital pain without fever, nausea, or vomiting one day later. Therefore, he was re-admitted for the fourth time, and lumbar puncture showed that the CSF pressure had increased to 300 mmH_2_O. The contrast-enhanced cranial MRI taken on July 6 showed a decrease in the lesion size. Thus, daily 8 mg methylprednisolone was resumed and daily 800 mg fluconazole was continued for anti-cryptococcal treatment. The symptoms of headache and orbital pain gradually subsided, and the patient was discharged on July 11. The patient continually received treatment at the local hospital, and voluntarily stopped using methylprednisolone on October 16. Currently, he is taking 800 mg fluconazole daily for cryptococcal infection,and now the patient has returned to the local hospital for folow-up.

## Discussion

Currently, there are scarce guidelines available on the management of previously healthy hosts who develop CM-PIIRS.Here we present a case of a CM-PIIRS, and evaluate the use of glucocorticoids based on his condition. This case report can explore treatment strategies and improve the prognosis of CM in immunocompetent individuals.

CM is a severe inflammatory mycosis of the central nervous system (CNS) caused by infection of the meninges or brain parenchyma with *Cryptococcus* species. *C. neoformans* and *C. gattii* are the primary etiological agents, and the most commonly involved sites are the CNS and lung [2]. Cryptococcosis caused by *C. neoformans* is prevalent across the country, and *C. gattii* infection is more common in the subtropical and tropical regions. *C. gattii* can be isolated from shrubs and other saprophytes, and is often detected in immunocompetent people without underlying diseases, with the lung being the most frequently infected site. Our patient is a logger and therefore at an increased risk of being infected with *C. gattii*. Furthermore, the endemic area of *C. gattii* infection has gradually expanded, and several reports of its involvement in the CNS have emerged in recent years [2, 3]. Meningoencephalitis due to *C. gattii* has been reported in China, which is more likely to involve the CNS than *C. neoformans*. In addition, it frequently shows an acute onset, severe symptoms and poor prognosis [4–6].

The patient initially presented with fever, and gradually developed other clinical manifestations such as headache and projectile vomiting, indicating the presence of significant intracranial hypertension. The occlusion caused by cryptococcal capsular polysaccharides prevents the passage of CSF through the arachnoid membrane, resulting in severe intracranial hypertension that usually manifests as headache, vomiting, papilledema, decreased visual acuity, blindness, confusion, and coma [7]. High intracranial pressure is difficult to treat due to the risk of brain herniation and poor prognosis. Therefore, the timely and effective control of intracranial hypertension is one of the most critical factors determining the outcome of CM [8]. Treatment methods include drugs such as mannitol, glycerin fructose, and furosemide, along with multiple invasive procedures, such as lumbar puncture for CSF drainage, Ommaya reservoir implantation, external ventricular drainage, and ventriculoperitoneal shunt [9]. We performed lumbar puncture several times, and also administered mannitol and other drugs to control intracranial hypertension, which increased the time window for successful anticryptococcal therapy.

Persistent infection and recurrence are the major challenges in CM treatment. Persistent infection is defined as the presence of *Cryptococcus* in CSF culture after four weeks of administering antifungal agents at effective doses. Recurrence refers to the change in the status of CSF culture from negative in response to treatment to positive, which coincides with the reappearance of the symptoms and signs of infection [10, 11]. Our patient showed deterioration of symptoms following several periods of improvement during the treatment regimen. Clinical symptoms, CSF pressure, and biochemical tests suggested significant remission after the patient was transferred to our department, and several CSF cultures were negative for *Cryptococcus*. However, the patient developed headaches, projectile vomiting, increased intracranial pressure, and new intracranial lesions. These symptoms were similar to that of immune reconstitution inflammatory syndrome (IRIS) in HIV-negative patients, also known as PIIRS [2].

Only a few studies have been conducted on CM-PIIRS, and there is no universal diagnostic criteria or a standardized treatment protocol. We diagnosed CM-PIIRS based on abnormal deterioration of clinical symptoms, changes in cranial MRI, and variations in relevant inflammatory parameters in CSF after effective antifungal therapy (persistently negative fungal culture), after excluding active infections, tumors, and drug causes [2, 12]. *C. gattii* utilizes the neurotransmitters (including dopamine, epinephrine, etc.) in the CSF, which contributes to the formation of melanin, protection against oxidative stress and phagocytosis, and development of CM-PIIRS [13]. There are reports that low-dose glucocorticoids can significantly improve symptoms of CM-PIIRS [12, 14]. Therefore, we treated the patient with sodium phosphate dexamethasone and achieved a satisfactory effect. However, there is no uniform standard regarding the dose, course of treatment and withdrawal time of hormones during subsequent treatment. Previous reports indicate that patients with CM-PIIRS can be intravenously injected with 1 g methylprednisolone daily for seven days, and then switched to 1 mg/kg of oral prednisolone daily for one month. The dose of prednisolone can be reduced by 5 mg per month according to the clinical manifestations and cranial MRI changes [14]. Kulkarni et al. reported the case of CM-PIIRS wherein 1 mg/kg oral prednisolone was given daily for seven days, gradually reduced, and then finally discontinued within two months [15]. The patient was discharged after clinical symptoms improved dramatically. In our case, the patient's condition gradually improved after receiving glucocorticoids and anticryptococcal therapy, and CM did not recur during glucocorticoid reduction. However, self-discontinuation of glucocorticoids led to recurrence of the symptoms, which subsided after the initial dose was resumed. Therefore, additional research is required to better understand the use of glucocorticoids.

CM often occurs in AIDS patients and *C. neoformans* is the most common etiological agent. However, the incidence of non-AIDS-related CM has increased significantly in recent years, which increases the risk of misdiagnosis and mistreatment [16], especially for *C. gattii* meningoencephalitis [4]. Furthermore, *C. gattii* infection shows a regional distribution. Several studies have reported that the combination of amphotericin B, flucytosine and fluconazole can effectively treat CM in the initial stages [17]. More clinical studies are needed to ascertain whether *C. gattii* meningoencephalitis can be treated with this regimen, given its acute onset, severe disease progression, and poor prognosis. The emergence of IRIS during the treatment of AIDS-related CM is well-documented, and the development of CM-PIIRS in the HIV-negative patients with CM is also worthy of attention. It is worth investigating whether anti-cryptococcal glucocorticoid therapy can prevent CM-PIIRS, and the dosage, duration, and timing have to be standardized in order to improve prognosis.

## Conclusion

The incidence of *C. gattii* meningoencephalitis has increased in HIV-negative patients in recent years. The combination of two anti-cryptococcal drugs, CSF drainage with therapeutic lumbar puncture, management of intracranial hypertension with mannitol and other drugs, and early identification and symptomatic treatment of CM-PIIRS can improve patient outcomes. This case report provides new insights into the diagnosis and treatment of CM in HIV-negative patients, which can improve prognosis.

## Data Availability

All data and materials in this article are included in the manuscript.

## Abbreviations

CM: Cryptococcal meningitis
HIV: Human immunodeficiency virus
PIIRS: Post-infectious inflammatory response syndrome
CSF: Cerebrospinal fluid
WBC: White blood cell
Hb: Hemoglobin
PLT: Platelet
CRP: C-reactive protein
PT: Prothrombin time
PCT: Procalcitonin
ALT: Alanine aminotransferase
AST: Aspartate aminotransferase
AKP: Alkaline phosphatase
GGT: Glutamyl transpeptidase
HBDH: α-Hydroxybutyrate dehydrogenase
CK: Creatine kinase
LDH: Lactate dehydrogenase
CNS: Central nervous system
IRIS: Immune reconstitution inflammatory syndrome
